# Influence of Combined Effect of Ultra-Sonication and High-Voltage Cold Plasma Treatment on Quality Parameters of Carrot Juice

**DOI:** 10.3390/foods8110593

**Published:** 2019-11-19

**Authors:** Muhammad Umair, Saqib Jabbar, Ahmed M. Senan, Tayyaba Sultana, Mustapha M. Nasiru, Assar A Shah, Hong Zhuang, Zhang Jianhao

**Affiliations:** 1 National Center of Meat Quality and Safety Control, Synergetic Innovation Center of Food Safety and Nutrition, College of Food Science and Technology, Nanjing Agricultural University, Nanjing 210095, Jiangsu, China; umair_uaf@hotmail.com (M.U.); Ahmedmsenan@njau.edu.cn (A.M.S.); 2018208036@njau.edu.cn (M.M.N.); 2 Food Science Research Institute (FSRI), National Agricultural Research Centre (NARC), Islamabad 44000, Pakistan; saqibjabbar2000@yahoo.com; 3 College of Public Administration, Nanjing Agriculture University Nanjing, Nanjing 210095, Jiangsu, China; 2015209032@njau.edu.cn; 4 National Forage Breeding Innovation Base, Institute of Animal Science, Jiangsu Academy of Agricultural Sciences, Nanjing 210014, Jiangsu, China; drshah290@yahoo.com; 5 Quality & Safety Assessment Research Unit, USDA-ARS, Athens, GA 30605, USA; hong.zhuang@ars.usda.gov

**Keywords:** combined effect, high voltage atmospheric cold plasma, ultra-sonication, dielectric barrier discharge, quality parameters

## Abstract

Influence of the combined effect of ultra-sonication (US) and high-voltage cold plasma treatment (HVCP) on the quality parameters of fresh carrot juice has been studied. During the treatment of ultra-sonication, carrot juice was subjected to a 0.5 inch probe for 3 min by adjusting the pulse duration 5 s on and off at 20 kHz frequency, amplitude level 80%. The ultrasound intensity was measured by using a thermocouple and was 46 Wcm^−2^. The temperature was maintained at 10 °C by an automatic control unit. During the treatment of HVCP, carrot juice was then subjected to dielectric barrier discharge (DBD) plasma discharge at 70 kV voltage for 4 min. Significant increases were observed when HVCP treated carrot juice was tested against total carotenoids, lycopene, and lutein when compared to the control treatments. Moreover, this increase was raised to its highest in all pigments, chlorogenic acid, sugar contents, and mineral profile, as the results of ultra-sonication when combined with high voltage atmospheric cold plasma (US-HVCP). Whereas, a significant decreased was observed in Mg, total plate count, yeast, and mold after US-HVCP treatment. Furthermore, results indicated that the combined effect of US-HVCP treatment has improved the quality and led to a higher concentration of lycopene, lutein, chlorogenic acid, and mineral compounds (Na, K, and P). Therefore, the findings of the current study suggested that US-HVCP treatment is a novel combined technique that could provide better quality and more stability during the processing of carrot juice with better physicochemical properties and bio-available nutrients, so this novel processing technique could serve as an alternative to traditional processes.

## 1. Introduction

Carrot (Daucus carota L.) of the Umbelliferae family belongs to an essential root vegetable. It is grown in many parts of the world; China is the leading country across the globe in carrot production [1]. Carrots are commonly eaten as fresh vegetable but due to their perishable nature, they are processed into a variety of products including juices. Carotenoids, bio-active compounds, vitamins, and minerals are derived from carrot and its products. These products are used because they can provide numerous health benefits to the human body [2]. During storage, undesirable browning reactions occur, which ultimately cause discoloration of the product due to the condensation of phenolic compounds [3]. Carrot juice color and flavor is an important quality indicator that attract customer and fulfill customer demand [4].

Blanching is a principal method employed in the processing of fruits and vegetables to preserve color and inhibit the growth of microorganisms [5]. Despite the benefits of blanching, it also has a negative impact on texture, water-soluble, heat-sensitive nutrients, which is due to the heat applied during processing [6,7]. 

Nowadays, many functional compounds from plant materials have been successfully extracted by using non thermal processing technologies such as microwave-assisted extraction (MAE), irradiation (UV), ultrasound-assisted extraction (UAE), ultra-sonication, high hydrostatic pressure (HHP), modified atmospheric pressure (MAP), pulse electric field (PEF), and ultra-sonication (US). All of the above-mentioned techniques have some limitations such as limited effect, high processing and equipment cost, and strict operating conditions [8,9].

On contrary, high voltage cold plasma non-thermal treatment (HVCP) is a new kind of non-thermal sterilization technique that has certain benefits and merits than traditional solvent extraction and processing techniques such as non-thermal and preserves heat-sensitive products that is gaining more attraction and importance as an alternative to heat treatment. Furthermore, equipment operation is simple, and there is no involvement of water, therefore, it can be widely used in the food industry [10,11,12,13,14]. Moreover, changes in the final product after treatment with HVCP should be considered for its practical implementation [15]. Some reports have shown that HVCP has no effect on color, particularly on lightness L* [16]. HVCP-treated pomegranate juice also exhibited a reduction in a* and b* values [17]). However, there were observed reductions in L* and b* values with a concomitant slight increase in a* value after the treatment of apple juice with an atmospheric pressure plasma (APPJ) jet [16]. Although a full biochemical mechanism that describes this increase is still unknown, it has been previously reported that ionized nitrogen species reacts to breaking bonds between sugars and cell membranes. This could be attributed to electrically charged species from the cold plasma, which may result in the electroporation of the cell–matrix and alter the hydrophobic or hydrophilic nature of the membrane and promote extraction ability as explained earlier [18]. Furthermore, the other potential benefits of HVCP treatment are reduced processing time, high throughput with low energy input, removal of solvent amount, and is environmentally friendly [19].

In recent years, the expectancy of the combinative effect of these non-thermal techniques in quality enhancement and reduction in the quality deterioration of fruit juices have been extensively studied [10,12,13]; a combination of two non-thermal techniques, pulse electric field (PEF) and US, has shown its capability to augment the safety and quality of fruit juices with minimal nutrient losses [20]. Furthermore, proved their potential to attenuate the microbial load in juices has been proven besides increasing the shelf life of the product. The key benefit associated with the use of combined techniques is the improved nutritive value of food products due to the less thermal degradation of heat-sensitive nutrients. Additionally, non-thermal food processing [21] techniques also provide microbial safety to the food without thermal degradation [22]. Furthermore, the other potential benefits of this novel method are reduced processing time, less energy inputs, high throughput, and it is environmentally-friendly [19]. This perspective of overcoming the existing conventional physical and chemical methods for sterilization makes it a novel technique to keep food with its natural nourishment, composition, appearance, structure, and freshness [23]. 

Therefore, the proper extraction method is necessary to obtain the maximum recovery of bio-active compounds with more stability from fresh carrot juice. The product can be treated with HVCP and US, which offers a better retention of heat-sensitive nutrients along with its desirable nutrient quantity and quality that are normally lost during blanching, heating, sterilization, and irradiation. However, no scientific data on the combination of HVCP and US are available as it its use as a novel non-thermal method. Accordingly, the aim of the present work is to explore the effect of US-HVCP as a novel technique on the different quality parameters of carrot juice. This is potentially a first study to report the combined effect of HVCP and US treatment on total carotenoids, β-carotene, lycopene, and ascorbic acid with some physicochemical properties and microbial stability of carrot juice.

## 2. Materials and Methods

### 2.1. Procurement of Different Chemicals 

Chemicals such as lutein, chlorogenic acid, sugars (β-glucose, sucrose, and fructose), β-carotene, and pectin were bought from Sigma Aldrich Chemie GmbH (Steinheim, Germany). Sucrose was purchased from Fluka Chemie GmbH (Buchs, Switzerland). Citric acid, butylated hydroxytoluene (BHT), formic acid, acetonitrile, and acetone were purchased from Sinopharm Chemical Reagents Co. Ltd. (Shanghai, China). Methanol and acetone (HPLC grade) were obtained from Hanbon Science and Tech. (Jiangsu, China). Sodium chloride, sodium sulfate, and sodium hydroxide were purchased from Xilong Chem. Factory, (Shantou, China). Nitric acid, n-hexane, hydrogen peroxide (H_2_O_2_), and petroleum ether were provided by Nanjing Chem. R. (Jiangsu, China).

### 2.2. Purchasing of Raw Material Blanching

Fresh carrots (*Daucus carota* cv. Heitian-5) were purchased from a local vegetable market in Nanjing, China. After purchase, carrots were treated within 24 h. The same batch of carrots was used for all treatments. Good quality carrots (1 kg) were washed using tap water, peeled, and then sliced manually to a thickness of approximately 2 cm with a diameter of 10 mm. After, the sliced carrots were pressed to make juice by using a Hurom slow juicer (Model# HU7WN3L). One part of fresh carrot juice was selected as the control without any treatment while the other parts of the carrot juice was subjected to HVCP and US treatments as designed and explained in the experiment.

### 2.3. High Voltage Cold Plasma and Ultra-Sonication Treatments 

**Figure 1:**
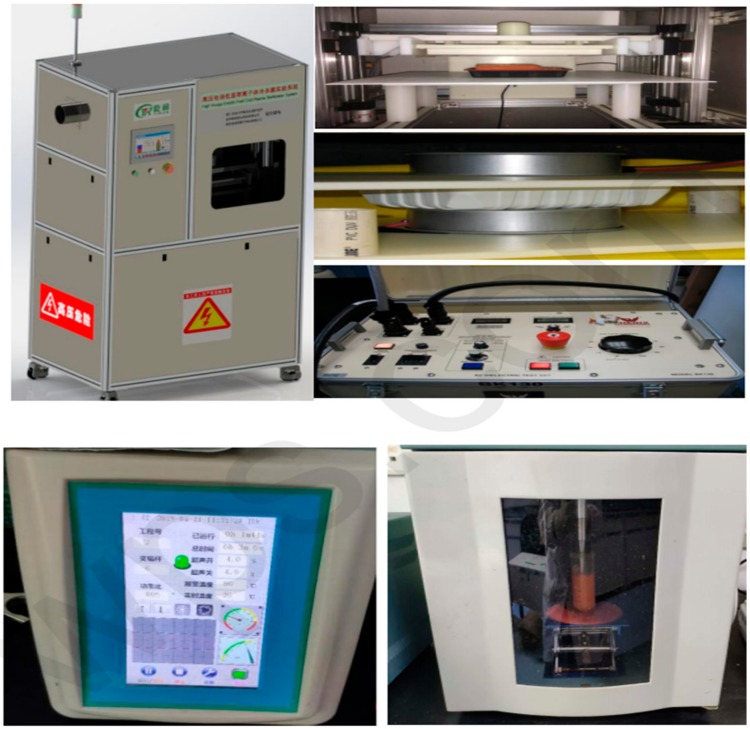
Schematic diagram of the ultra-sonication in combination with high voltage cold plasma (US-HVCP) treatment of carrot juice.

A preliminary trial was conducted to evaluate the optimal conditions for HVCP and US treatments. Freshly prepared carrot juice was then treated using HVCP. A dielectric barrier discharge (DBD) plasma source was used in this study as described by Wang et al. [24], which consisted of a reaction cell (DBD-50), a high voltage alternating current power source (CTP-2000K), a voltage-regulator (Nanjing Suman Electronics Co., Ltd., Nanjing, Jiangsu, China), and a quartz dish (with an outer diameter of 70 mm, inner diameter of 50 mm, a wall thickness of 9 mm, and a depth of 4 mm). The quartz dish was placed between the two quartz plates at a 70 kV voltage for 4 min. The schematic diagram can be seen in Figure 1. HVCP treatment was applied in a dark room to reduce external interference. Each sample was treated three times for 4 min with a 30-second rest interval between each 4 min of treatment. All samples were treated in triplicate for statistical analysis to reduce the relative error of the treatments. The sample was packed in sterilized food grade packed bottles during HVCP treatment. All treatments were repeated three times. A fresh untreated carrot juice sample was taken as a control sample. After HVCP treatment, the carrot juice was subjected to US treatment. 

Carrot juice samples were treated for US treatment at 15 °C for 3 min with each 5 s pulse on and off-cycle. Approximately, 100 mL of juice sample was taken in a 500 mL jacketed vessel (7.6 cm ID × 9.3 cm OD × 13.5 cm depth × 14.8 cm height). Temperature was controlled by the circulation of cold water around the jacketed vessel. The temperature rise in carrot juice with US treatment was 2–4 °C. US treatment of carrot juice was performed with the help of an ultrasonic processor (VC-750; Sonics and Material Inc., Newtown, CT, USA) at a frequency of 20 kHz with the amplitude level of 80% with a 0.5-inch probe and 2 cm depth in the sample. The schematic diagram of the exposure is shown in Figure 1. The ultrasound intensity was measured by a thermocouple (HI-9063 Hanna Instruments Ltd., Buzdar, UK) by using Equation (1) [25]. All treatments were repeated three times. A fresh untreated carrot juice sample was taken as a control sample.

(1) $$I=\frac{\mathrm{mCp}}{A}\left[{\left(\frac{\mathrm{dT}}{\mathrm{dt}}\right)}_{a}-{\left(\frac{\mathrm{dT}}{\mathrm{dt}}\right)}_{b}\right]$$

where I is the ultrasound intensity and m is the mass of sample; C represents the heat capacity; A is the surface area of the sonicator probe; (dT/dt)_a_ represents the slope of the initial temperature increase; and (dT/dt)_b_ is the heat loss after the reactor was switched off. 

### 2.4. Determination of Total Carotenoids Content

Total carotenoids were determined in all treatments by using the method described by Lieu et al. [26] with a minor change. A total of 25 mL of the control and treated samples were taken in a separating funnel and 80 mL of mixture solution (n-hexane/acetone 1:1, *v*/*v*) was added into the separating funnel and shaken well until the red color of the carrot juice disappeared. Sodium sulfate was added to dehydrate the organic phase of the solution in the separating funnel. A UV–Vis spectrophotometer (Shimadzu Scientific Instruments Co. Ltd., Tokyo, Japan) was used to measure the absorbance at 450 nm to measure total carotenoids. Commercially available β-carotene was purchased as an external standard and solutions of different concentrations (2–10 µg/mL) of β-carotene were prepared. Data were analyzed as mg of β-carotene equivalent per mL of carrot juice.

### 2.5. Determination of Lycopene

All of the treated samples and the control were tested to determine the effect of HVCP on lycopene contents. Lycopene was determined by following the method by Oliu et al. [7] with minor modification. As mentioned below, the juice sample (0.6 mL) was mixed with 20 mL of reaction reagent and centrifuged by an Eppendorf centrifuge 5804R (Eppendorf AG, Hamburg, Germany) for 15 min at 330× *g*. A total of 3 mL distilled water was added into the above-centrifuged mixture and left for another 5 min at room temperature for phase separation. The upper light yellow color n-hexane layer was used to measure changes in the absorbance at 503 nm through an UV–Vis spectrophotometer (Shimadzu Scientific Instruments Co. Ltd., Tokyo, Japan). A pure n-hexane solution was used as a blank. Equation (2) [27] was used to determine the lycopene contents in the carrot juice samples.

(2) $$\mathrm{Lycopene}=\;\frac{\Delta 503\times \mathrm{MW}\times \mathrm{DF}\times 1000}{\Sigma \times L}$$

where MW is the molecular weight of lycopene (536.9 g/mol); DF is the dilution factor; L is the path length in cm; and ε is the molar extinction coefficient for lycopene as reported earlier by Rawson [28]; and is 172,000 L/mol cm. Lycopene contents were expressed as mg/mL in all samples. All experiments were treated in triplicate

### 2.6. Determination of Lutein

All of the treated samples and control were tested to determine the effect of HVCP on lutein content. The Kim et al. [29] method with slight changes was used for carotenoid determination. First, the juice samples were extracted through the separating funnel with an acetone-based solvent (three times), then filtered using filter paper (Cat# GB/T 1914-2007, Whatman Filter International, Maidstone, England), and the filter scum was re-extracted using methanol. Petroleum ether with equal volumes was mixed with the above methanol–acetone extract. After filtration, anhydrous sodium sulfate was added to the upper petroleum layer to dehydrate it, then a rotary evaporator was employed to concentrate the solution. A sample of 10 mL of acetonitrile-acetone-methanol (40:20:40, *v*/*v*) solution was added and placed in a dark place until further usage.

The Kim et al. [29] method was used for lutein determination. The identification and quantification of lutein content were made through HPLC (Waters 600 system). A 20 µL sample was filtered through a syringe filter (0.45 μm, 13 mm Cat. # AS-021345 N-Agela Technologies) and fractioned by Waters Auto Purification HPLC with column OBD^TM^ C 18 (5 μm, 150 mm × 19 mm, Cat. #1501382671, X-Bridge^TM^, Dublin, Ireland). The mobile phase comprised of a mixture of acetonitrile: Acetone: Methanol (40:20:40, *v*/*v*), and the flow rate was adjusted to 0.80 mL/min. Commercially available lutein was used as an external standard. Different concentration solutions of standard lutein were used to draw a linear regression calibration curve.

### 2.7. Determination of Chlorogenic Acid

The method by Kahle et al. [30] with minor modification was used to determined chlorogenic acid. The identification and quantification of chlorogenic acid was through HPLC (Waters 600 system). The supernatants were filtered (0.45 μm, 13 mm Cat. # AS-021345 N-Agela Technologies) and fractioned by a Waters Auto Purification HPLC with column OBD^TM^ C 18 (5 μm, 150 mm × 19 mm, Cat. #1501382671, X-Bridge^TM^, Dublin, Ireland). The mobile phase consisted of a mixture of aqueous formic acid (A) with methanol (B) (0.1:99.9, *v*/*v*). A total of 5 mL of the filtrate sample was injected into the column. The linear biphasic gradient was used with 15–40% solvent B over 10 min, 40–50% over 15 min, 50–75% over 20 min, 75–90% over 25 min, 90–65% over 30 min, 65–40% over 35 min, and 40–10% over 40 min. The flow rate was adjusted to 1 mL/min throughout the 40 complete circles of 40 min. Elution was observed at a wavelength of 320 nm through UV detection. Commercially available chlorogenic acid was used as an external standard. Different concentration solutions (10–80 mg/mL) of chlorogenic acid were used to draw a linear regression calibration curve. Data were analyzed as mg of chlorogenic acid equivalent per mL of samples.

### 2.8. Determination of Sugar Content in Carrot Juice

Sugar content was determined by Hurst et al. [31]. The identification and quantification of sugar contents were performed using a semi-preparatory high-performance liquid chromatography (HPLC) system (waters 600). The supernatants were filtered (0.45 μm, 13 mm Cat. # AS-021345 N-Agela technologies) and fractionated by Waters Auto Purification HPLC. The mobile phase consisted of acetonitrile (75:25, *v*/*v*) with a 1 mL flow rate. A Cosmosil packed column of D-sugars (4.6 × 250 mm) was used. Commercially available sucrose, fructose, and glucose were used as an external standard. Different concentrations of each standard solution of sucrose, fructose, and glucose were used to draw a linear regression calibration curve. Data were analyzed as gram of sucrose, fructose, and glucose equivalent per liter of sample. 

### 2.9. Determination of Mineral Contents 

The American Public Health Association [32] described method was followed to determine the mineral contents. A total of 1 mL of the carrot juice sample was digested with 7 mL of nitric acid solution (65:35, *v*/*v*) in a Teflon vessel (TFM). All conditions for the detection of minerals are given below in Table 1. 

A total of 1 mL of H_2_O_2_ was added to this mixture and heated at 200 °C in a microwave oven for 20 min with a one-minute rest after the first ten min for better acid digestion. The digested solution was transferred into a 50 mL volumetric flask and diluted with pure water up to the 50 mL mark. The samples were tested using an inductively coupled plasma-optical emission spectrometer (OPTIMA^TM^ 2100 DV, Perkin Elmer Precisely, Shelton, CT, USA). Commercially available standards of each mineral were purchased and used as an external standard. All measurements were conducted in triplicate. 

### 2.10. Physicochemical Analysis 

#### 2.10.1. Determination of °Brix

°Brix was measured by a hand refractometer (WYT-J 0~32% Chengdu Haochuan Guangdian company, Chengdu, China) at room temperature (25 ± 2 °C). All measurements were taken in triplicate. Water was used to wash the prism of the hand refractometer. 

#### 2.10.2. Determination of pH in Carrot Juice 

A digital pH meter was used to measure changes of pH in the carrot juice samples. Different concentration buffer solutions (pH = 4.0 and 7.0) were used for the calibration of the pH meter. A total of 15–20 mL of each treatment sample was poured into a beaker and the changes of pH measured with the pH meter. All measurements were conducted in triplicate.

#### 2.10.3. Determination of Color Changes in Carrot Juice

The color was measured using a colorimeter (CR-400, Konica Minolta sensing Inc., Osaka, Japan) with the CIE Lab tristimulus parameters: L*, a*, and b*. The L* measures brightness/whiteness of color, a* describes the level of redness (+a*) or greenness (−a*), and b* is used to describe the level of yellowness (+b*) and blueness (−b*). A standard white plate (L* = 97.95, a* = 0.89 and b* = 1.23) was used to calibrate the instrument. There were nine samples and each piece was recorded three times. Moreover, the carrot juice was critically evaluated for Hue angle value as related to the intensity of red chromaticity; Chroma or saturation value degree of changes from gray to lightness; browning index; and total color differences. The following formulas can be applied to determine the Hue angle (h°), saturation value (C*), and browning index (BI) where x = (a* + 1.75 L) (5.64 L+ a* − 3.012 b*) and total color difference (ΔE) is explained in Equations (3)–(6) [33,34].

(3) $$\mathrm{Hue}\;(h\circ )=\left[\left(\frac{1}{\tan}\right)\times \left(\frac{b*}{a*}\right)\right]$$

(4) $$C*=\sqrt{{\left(a*\right)}^{2}+{\left(b*\right)}^{2}}$$

(5) BI= [100(X−0.31)] 0.17

(6) $$\Delta E=\sqrt{{(\Delta L*)}^{2}+{(\Delta a*)}^{2}+{(\Delta b*)}^{2}}$$

### 2.11. Total Plate Count (TPC)

Bacteriological analyses of treated and control carrot juice samples were carried out by following the Food and Drug Administration (FDA) analytical manual [35]. The total plate count (TPC) was performed by the pour plate technique. Briefly, distilled water was used to prepare serial dilutions (up to 10^−5^) of the samples. A total of 1 mL of sample was transferred into sterilized Petri plates, then 20 mL of PCA agar was poured into each Petri plate containing 1 mL of each diluted treatment and control sample. Mixing was done gently (by moving clockwise and anticlockwise) to mix the sample uniformly throughout the plates. The Petri plates were then allowed to grow in an incubator (GSP-9080 MBE, Shanghai Boxun Industry & Commerce Co. Ltd., Shanghai, China) at 37 °C for at least 24 h. Bacterial colonies were counted in juice samples as CFU/mL of carrot juice and expressed as log CFU/mL of the sample.

### 2.12. Yeast and Mold Counts

Pour plate technique was used to explore the effect of high voltage cold atmospheric plasma (HVCP) on the growth of yeast and mold [35]. PDA agar was used for the purpose of growing yeast and mold in Petri dishes; tartaric acid (10:90, *w*/*v*) was added during the preparation of PDA agar. A total of 1 mL of the sample was inoculated into the Petri dish and then 15–20 mL of PDA agar was poured into a Petri dish and mixed gently for 2 min to disperse the sample throughout the plate, before the plates were placed in an incubator at 28 °C for 48 h. Yeast and mold counts in carrot juice samples are expressed as CFU/mL of juice. All experiments were carried out in triplicate.

### 2.13. Statistical Analysis

The mean values and standard deviation of the results were calculated. Complete randomized design (CRD) was applied with one-way ANOVA to determine the level of significance (*p* < 0.05). All significant values between means were determined by the least significant difference (LSD) pair-wise comparison test. The data were analyzed using SPSS 18.0 (IBM Inc., Armonk, NY, USA) for Windows.

## 3. Results and Discussion

### 3.1. Estimation of Chlorogenic Acid 

Data regarding the impact of HVCP and other treatments are listed in Table 2. Results indicated that there was a significant increase in chlorogenic acid in the case of HVCP and the US when compared with the control. However, this increase was more significant in the case of the combined US-HVCP treatment when compared to the control. This increase could be due to the better extraction capacity and maximum disruption of the cell structure mechanism, which results in a better-releasing capacity of cell-bound chlorogenic acid. This was also explained by other studies when the combined effect of ultra-sonication and blanching was measured for carrot and apple juices [25,36]. Thus, the application of HVCP with US is a new and emerging process and a technique with more beneficial attributes as it shows the potential to better recover/improve the contents of chlorogenic acid in carrot juice that are lost during traditional blanching treatment for carrot juice processing.

### 3.2. Estimation of Coloring Compounds

#### 3.2.1. Determination of Total Carotenoids

Carotenoid is the key component of carrot juices and other carrot products and mainly responsible for the red color and gives better eye appeal. Carotenoids have been shown to have a potentially strong antioxidant capacity in previous studies [27,37]. The findings in this study showed that US-HVCP improved the total carotenoids in carrot juice when compared to the control. The different treatment effects on the extraction and determination of total carotenoids is explained in Table 2. The red-color retention in carrot juice is mainly due to better preservation of carotenoids during the juice processing and this has also been observed in HVCP and US, but this retention was highly significant in US-HVCP when compared to all other treatments. This better and efficient pigment (carotenoid) preservative effect of US-HVCP may be due to the effective inactivation of enzyme activities that are mainly because of combining the effect of this novel processing tool by using ultra-sonication and HVCP. There have been some reports that have also explained that the combined effect of two non-thermal techniques could enhance the severity of the effect than that of these techniques separately [8,27,34]. Therefore, the combining effect of US with HVCP could serve as a potential processing technique for better quality extraction, preservation, and storage of carrot juices.

#### 3.2.2. Effect of HVCP and US on Lycopene and Lutein Contents

Lycopene is another key component in all red color fruits and vegetables and has significant importance to consider during the processing conditions. The combined effect of different treatments on lycopene and lutein has been explained in Table 2. From the data, it is clear that all of the HVCP and US treated samples had a better retention of lycopene and lutein contents when compared to fresh untreated juice samples. However, the highest increased was observed in US-HVCP. In some previous reports, it was also proven that lycopene and lutein contents were increased significantly when the US treated carrot juice was compared with normal untreated carrot juice [27]. Furthermore, a more prominent increase in the combined effect is also in accordance with previously conducted studies in which the effect of ultra-sonication in combination with blanching has been observed for apple, pear, and grapefruit juices [8,36]. This better retention of lycopene and lutein contents in the blanched sample is due to the weakening of binding forces between the tissue matrix and cell wall, which leads to the breakdown of cell structure and improvement in cis-isomerization [38]. 

This phenomenon has also been explained by another scientist that the improvement of lycopene and lutein contents during processing is mainly due to the conversion of the trans-isomer of nutrients to a more bio-available (cis-isomer) form, which ultimately enhanced the availability of the lycopene and lutein in the solution [39]. Similarly, increases in lycopene and lutein contents were observed due to ultra-sonication that might be due to cavitational force and mechanical breakdown, which results in serious chromoplast membrane and cell wall damage [8,14,27,34]. This is, therefore, a very clear indication that the combined effect of the US with HVCP is a promising technique to improve the quality of processed juices by retaining and making its naturally occurred nutrients more bio-available.

### 3.3. Determination of Sugars Contents in Processed Carrot Juice 

This study explained the effect of different treatments on the sugar contents of the processed carrot juice. The results show that there was a significant increase in sugar contents (sucrose, fructose, glucose) in all samples treated with HVCP, US, and US-HVCP when compared to the control. 

The results of the combined effect of the US and HVCP are listed in Table 3. This increase was perhaps due to a better and sufficient capacity to disrupt sugars and cell–matrix. These results were also proven by previously reported studies, in which the effect of blanching on the sugar contents has been found for beetroot, carrots, and turnips [40]. While in the case of the US-HVCP sample, there was a significant increased. This increase in the contents of sugars can be attributed to intra-cellular sugars that are released into solution due to high voltage treatment, which causes a complete breakdown of the cell–matrix. This breakdown is further supported by the cavitational effect of US treatment, which releases intra-cellular sugars in the liquid when treated with HVCP and US.

Furthermore, the sugar contents were more significant in the case of US-HVCP when compared to other treatments. This phenomenon regarding the increase in sugar content in the US-HVCP carrot juice is in agreement with many previous reports [8,27,36,41]. Hence, the combined effect of US treated samples with high voltage cold plasma (US-HVCP) could enhance and improve the level of sugars in carrot juice, which justifies the practical application and industrial implementation of this combined treatment for juice and other liquid processing.

### 3.4. Impact of HVCP and US on the Mineral Profile of Carrot Juice 

**Figure 2:**
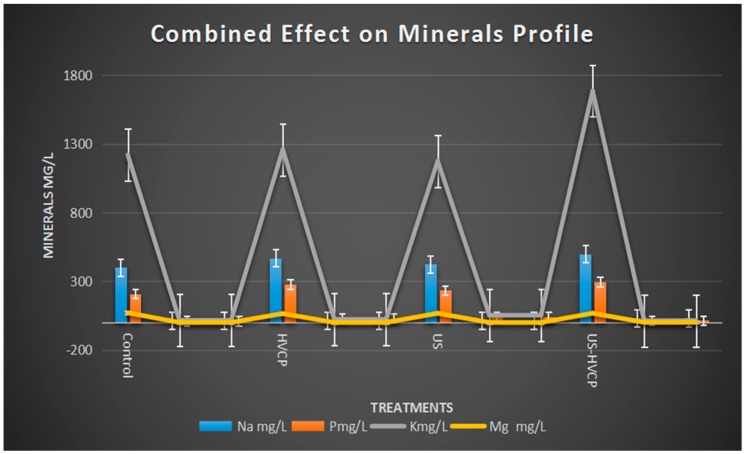
Combined treatment effect of US and HVCP on the mineral profile of the carrot juice.

The results regarding the combined effect of US and HVCP on the mineral profile (Na, P, K, and Mg) are demonstrated in Figure 2. A significant reduction was observed in the mineral profile (Mg) when treated with US treatment while significant increases in Na, K, and P were observed in HVCP, US, and US-HVCP samples when compared to the untreated controlled sample. A similar effect on mineral content (Na) was reported previously when the effect of ultrasound treatment on the quality of egg yolk was observed [42]. A similar declining trend was monitored for Mg when ultra-sonication treatment was applied for apple and carrot juices [27,34]. Conversely, the findings of the current study regarding the above were opposite to the previous study [42]. Therefore, further research is needed to explore the exact mechanism that explains the relationship between HVCP treatment with mineral profile analysis. For this reason, an increase in Na, K, and P in all of the high voltage treated samples is also a solid reason to consider and verify the combined effect of ultra-sonication and high voltage cold plasma for the processing of carrot juice.

### 3.5. Combined Treatment Effect of HVCP and US on Physico-Chemical Properties

Combined impact of HVCP and US on pH, °Brix, color index (L*: Lightness; a*: Redness; b*: Yellowness), Hue angle value, Chroma value or saturation index has been determined in carrot juice (Table 4). No significant changes were observed in pH and °Brix when treated with HVCP, US, and US-HVCP. These findings are as per a previous report [43]. While HVCP treatment improved color values significantly (L*, a*, and b*), a significant decrease was observed in the color values when samples were treated with US treatments. However, in the case of the combined treatment of US-HVCP, L* and b* values were decreased whereas the a* value was significantly increased. This phenomenon was also observed when PEF treatment was used to process apricot nectar [44] where the author reported that after PEF treatment, there was a decrease in the L* value and an increase in a* value was observed. This is might be due to carotenoid isomerization or the production of free radicals during US treatment as reported earlier [45]. Changes in color might be due to the activities of enzyme, which results in enzymatic browning. A small reduced effect of color value was observed in the case of US-HVCP and this was due to the lesser activation of polyphenol oxidase enzyme (PPO), therefore, a significant color retention effect was observed as a result of combined treatment. Changes in Hue angle and Chroma values have been reported as a tool to predict color with or without L*, a*, wand b*. 

Hue value was reported by Hue angle (h°). Data showed that hue angle increased in samples treated with HVCP, US, and US-HVCP when compared to the control, which corresponded to a redder hue during the treatment. However, this difference was more prominent in the combined treatment of ultra-sonication and high voltage cold plasma. This Hue value was found to be much lower in the control than that of the US-HVCP treatment, which indicated that the shift in Hue to a deeper red was associated with the combined treatment effect of US-HVCP. A similar finding was noticed in the Hue value of grapefruit juice when it was correlated with visual color scores [8]. The browning index (BI) of this study was also under the browning trend of the previous apple juice study by ultra-high pressure (HHP) with ultra-sonication [36]. However, in the case of the Chroma index, which is a function of saturation index, it expresses the shift of departure from gray to lightness. Minimal changes occurred in Chroma value in all treatments when compared to the control sample. This important quality factor also depicted the efficient inhibition of enzymes during the US-HVCP treatment, as a result, no significant increase in the degree of polymerization of browning pigments occurred, so no difference in the value of C* was noticed. The changes in the carrot juice color (ΔE) value also explained the overall improvement and superiority of the US-HVCP treatment. Therefore, we suggest that US-HVCP can be a novel non-thermal process for juice and other beverages.

### 3.6. Antimicrobial Activity Assay

**Figure 3:**
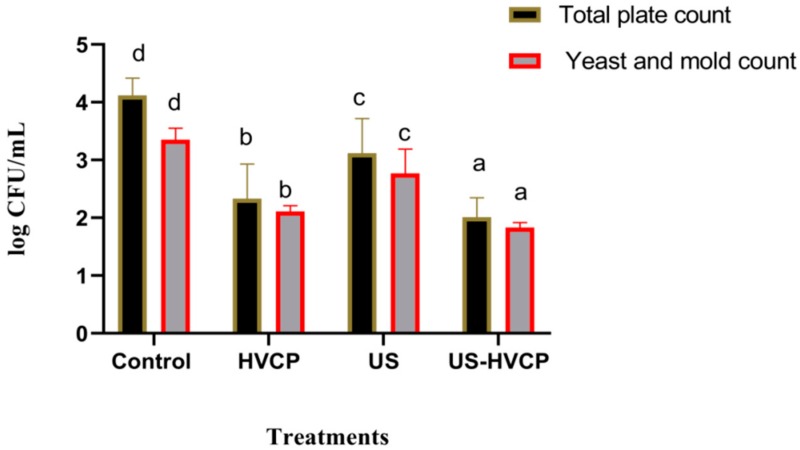
Combined treatment effect of US and HVCP on the microbial activity of carrot juice.

This factor is a key important factor for any processing technology. Without significant success in reducing and controlling microbes during processing, no technology can be authenticated. Therefore, this study also explained the combined effect of US and HVCP on antimicrobial activities and the results are explained in Figure 3. Total plate count (TPC) and yeast and mold counts were also tested in carrot juice. A significant reduction was observed in all US and HVCP treated samples when compared to the control untreated sample. US-HVCP showed the highest reduction in TPC when compared to other treatments. Similarly, a significant reducing effect of all US and HVCP treatments was observed while US-HVCP was prominent. Similar findings have been observed by other authors [8,27,34] who reported similar trends for microbial inhibition in their studies when they tested the combined effect of ultra-sonication and blanching for carrot, apple, and grapefruit juices. This better reduction in microbial activities in the US-HVCP treatment could be attributed to the production of reactive nitrogen and oxygen species (RONS) during the exposure of plasma generated atmosphere, and because the production of different reactive species such as ROS and RNS and the biocide effect of these reactive species could inhibit the growth of microorganisms and enhance it stability and shelf life. This also follows other previous reports in which the effect of gas-phase surface discharged plasma with a spray reactor for the *Zygosaccharomyces rouxii* inactivation in apple juice has been studied [46]. From all of the above findings and detailed discussion, it is clear that the combined effect is much more significant in improving the quality and safety of carrot juice and has very strong potential for practical application in juice and other beverage industries. Furthermore, it has been proven to reduce the microbial load and enhance the stability of carrot juice with better nutritional value and many more beneficial attributes that urge its industrial application for the cold processing of delicate food items, especially for those treatments that require saving heat losses with almost no side effects. Finally, considering all of the above-mentioned and its certain benefits such as a much simpler and controllable equipment handling and operation process, non-thermal, preserves heat-sensitive and water-soluble nutrients, has no involvement of solvents, and some other merits than that of traditional processing and preservation techniques, so we suggest that the combined treatment of ultrasonic treatment with high voltage cold plasma may successfully be employed and can be widely used in the food industry.

## 4. Conclusions 

From all of the above findings and detailed discussion, it is clear that the combined effect is much more significant in improving the quality and safety of carrot juice and that it has very strong potential for practical application in juice and other beverage industries. Furthermore, it has been proven to reduce microbial load and enhance the stability of carrot juice with better nutritional value and many more beneficial attributes that urge its industrial application for the cold processing of delicate food items, especially for those treatments that require saving heat losses with almost no side effects. Finally, keeping all considerations and its certain benefits such as a much simpler and controllable equipment handling and operation process, non-thermal, preserves heat-sensitive and water-soluble nutrients, there is no involvement of solvents, and some other merits than that of traditional processing and preservation techniques, so we suggest that the combined treatment of ultrasonic treatment with high voltage cold plasma may successfully be employed and can widely be used in the food industry.

## Figures and Tables

**Table 1:** Conditions of plasma-optical emission spectrometer.

S. No	Working Conditions	Flow Rate/Units
1	Elements (Na, P, K and Mg)	589.5, 213.6, 766.5 and 285.2
2	Nebulized gas discharge	0.85 L/Min
3	Plasma gas discharge	16.5 L/Min
4	Auxiliary gas discharge	0.21 L/Min
5	Plasma gas discharge	15 L/Min
6	Sample flow rate	1.8 mL/Min
7	Operating power	1450 W
8	View Axial	Interface shear gas
9	Sample uptake rate	1.25 mL/min
10	Spray chamber	cyclonic
11	Nebulizer type	Meinhard
12	Nebulizer set up	Instant
13	Replicates	3 times

**Table 2:** Combined effect of treatments on different quality parameters.

S. No	Treatment	Chlorogenic Acid µg/mL	Total Carotenoids µg/mL	Lycopene Contents µg/mL	Lutein Contents µg/mL
1	Control	22.30 ± 0.09 ^c^	8.22 ± 0.02 ^d^	0.52 ± 0.01 ^d^	1.22 ± 0.06 ^d^
2	HVCP	25.67 ± 0.08 ^b^	10.81 ± 0.03 ^b^	1.71 ± 0.09 ^b^	1.65 ± 0.05 ^b^
3	US	23.16 ± 0.02 ^b,c^	10.03 ± 0.08 ^b^	1.83 ± 0.05 ^c^	1.56 ± 0.09 ^c^
4	US-HVCP	27.31 ± 0.06 ^a^	11.03 ± 0.05 ^a^	1.93 ± 0.04 ^a^	2.03 ± 0.02 ^a^

Values with different letters (a–d) in same column represent that they are significantly different from each other. HVCP: High voltage atmospheric cold plasma (HVCP), US: Ultrasonic treatment, US-HVCP: Ultrasonic treatment and high voltage atmospheric cold plasma.

**Table 3:** Combined effect of blanching and HVCP on sugar content in carrot juice.

S. No	Treatment	Sucrose g/L	Fructose g/L	Glucose g/L
1	Control	41.04 ± 0.01 ^c^	18.65 ± 0.01 ^b^	20.52 ± 0.06 ^c^
2	HVCP	42.13 ± 0.03 ^b^	19.11 ± 0.02 ^a^	21.91 ± 0.02 ^a^
3	US	41.88 ± 0.06 ^b^	15.03 ± 0.03 ^b,c^	19.83 ± 0.04 ^c^
4	US-HVCP	42.36 ± 0.06 ^a^	16.03 ± 0.04 ^c^	21.23 ± 0.06 ^b^

Values with different letters (a–d) in same column represents that they are significantly different from each other. HVCP: High voltage atmospheric cold plasma (HVCP), US: Ultrasonic treatment, US-HVCP: Ultrasonic treatment and high voltage atmospheric cold plasma.

**Table 4:** Combined treatment effect of HVCP and US on pH, °Brix, and color indexes.

Treatment	pH	°Brix	Coloring-Index			Hue (h°)	C*	BI	ΔE
			L*	A*	B*				
Control	7.77 ± 0.20 ^a^	6.08 ± 0.01 ^a^	35.31 ± 0.24 ^a^	19.53 ± 0.22 ^a^	29.66 ± 0.10 ^a^	13.02 ± 0.03	21.05 ± 0.01	6.01 ± 0.62	0.24 ± 0.01
HVCP	7.77 ± 0.20 ^a^	6.08 ± 0.02 ^a^	36.83 ± 0.22 ^a^	18.58 ± 0.23 ^a^	31.59 ± 0.11 ^a^	13.22 ± 0.01	21.44 ± 0.03	7.89 ± 0.33	0.32 ± 0.01
US	7.77 ± 0.21 ^a^	6.08 ± 0.01 ^a^	38.72 ± 0.34 ^a^	20.83 ± 0.21 ^a^	32.95 ± 0.88 ^a^	13.25 ± 0.02	21.76 ± 0.06	8.11 ± 0.79	0.49 ± 0.02
US-HVCP	7.77 ± 0.21 ^a^	6.08 ± 0.01 ^a^	36.05 ± 0.12 ^a^	18.23 ± 0.20 ^a^	30.01 ± 0.21 ^a^	13.11 ± 0.01	21.14 ± 0.02	6.56 ± 0.02	0.27 ± 0.01

Values with different letters (a–d) in same column represent that they are significantly different from each other. HVCP: High voltage atmospheric cold plasma (HVCP), US: Ultrasonic treatment, US-HVCP: Ultrasonic treatment and high voltage atmospheric cold plasma.
